# Supplementary material for: Gold Nanoparticle Virus-like Particles Presenting SARS-CoV-2 Spike Protein: Synthesis, Biophysical Properties and Immunogenicity in BALB/c Mice
Source: Vaccines (Basel). 2024 Jul 23;12(8):829. doi: 10.3390/vaccines12080829 (PMC11359663; doi:10.3390/vaccines12080829)
Supplement: Supplementary file 1 [file vaccines-12-00829-s001.zip › vaccines-3083062-supplementary.pdf]

Supplementary Information

Gold Nanoparticle Virus-like Particles Presenting SARS-CoV-2 Spike Protein: Synthesis, Biophysical Properties and Immunogenicity in BALB/c Mice

Vivian A. Salazar [1], Joan Comenge [1,2], Rosa Suárez-López [3], Judith A. Burger [4], Rogier W. Sanders [4], Neus G. Bastús [2,5], Carlos Jaime [3], Joan Joseph-Munne [1,6,*] and Victor Puntes [1,2,5,7,*]

1. Vall d'Hebron Institut de Recerca, 08035 Barcelona, Spain; vivianangelica.salazar@icn2.cat (V.A.S.); joan.comenge.ext@vhir.org (J.C.)
2. Networking Research Centre for Bioengineering, Biomaterials and Nanomedicine (CIBER-BBN), Instituto de Salud Carlos III, 28029 Madrid, Spain; neus.bastus@icn2.cat
3. Departament de Química, Universitat Autònoma de Barcelona, Bellaterra, 08193 Barcelona, Spain; rosa.suarez@uab.cat (R.S.-L.); carlos.jaime@uab.cat (C.J.)
4. Department of Medical Microbiology and Infection Prevention, Amsterdam University Medical Centers, Location AMC, University of Amsterdam, Amsterdam Infection & Immunity Institute, 1105 AZ Amsterdam, The Netherlands; j.a.burger@amsterdamumc.nl (J.A.B.); r.w.sanders@amsterdamumc.nl (R.W.S.)
5. Institut Català de Nanociència i Nanotecnologia (ICN2), CSIC and BIST, Campus Universitat Autònoma de Barcelona, 08193 Barcelona, Spain
6. Department of Microbiology, Hospital Universitari Vall d'Hebron, 08035 Barcelona, Spain
7. Institució Catalana de Recerca i Estudis Avançats (ICREA), 08010 Barcelona, Spain

* Correspondence: joan.joseph@vallhebron.cat (J.J.-M.), victor.puntes@vhir.org (V.P.)

Molecular dynamics

Charge distribution of S protein is depicted in **Figure S1**. CG beads were color-coded to reflect their binding strengths to the AuNP, as suggested by the charge distribution on the original amino acid sequence. Dark red beads (H) denote the highest binding affinity, followed by red beads (M), and blue beads (L) representing the least attractive interaction (**Figure S1**). The Au NP itself was formed by the aggregation of gold beads (Au), and the surrounding solvent was depicted by water (W) beads.

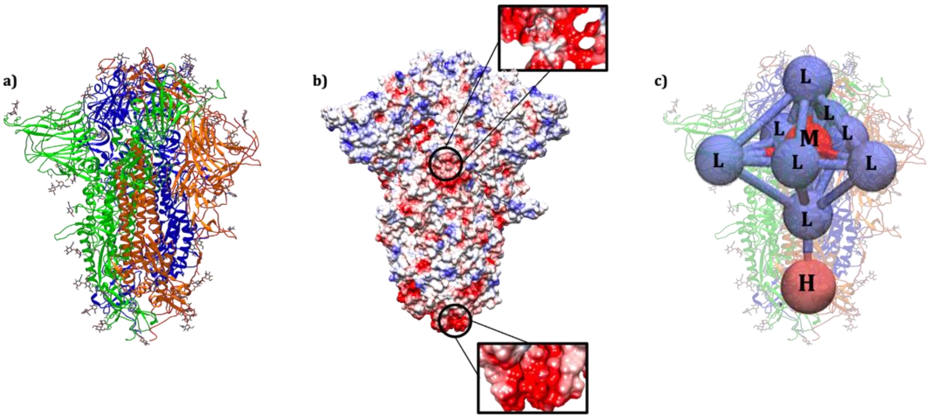

Figure S1. Comparison of S protein structures and binding affinity visualization. (**a**) All-atom structure of S protein. (**b**) Coulombic surface coloring generated using UCSF Chimera, with red spots amplified for clarity. (**c**) CG model highlighting beads based on their binding strength to AuNP; H (highest affinity), M (moderate affinity), and multiple CG beads (L) representing the least attractive interactions towards to AuNP.
